# Hypoxia enhances interactions between Na^+^/H^+^ exchanger isoform 1 and actin filaments *via* ezrin in pulmonary vascular smooth muscle

**DOI:** 10.3389/fphys.2023.1108304

**Published:** 2023-02-28

**Authors:** Julie M. Lade, Manuella R. Andrade, Clark Undem, Jasmine Walker, Haiyang Jiang, Xin Yun, Larissa A. Shimoda

**Affiliations:** ^1^ Division of Pulmonary and Critical Care Medicine, Department of Medicine, Baltimore, MD, United States; ^2^ Department of Physiology, Johns Hopkins School of Medicine, Baltimore, MD, United States

**Keywords:** smooth muscle, lung, smooth muscle actin, hypoxia, pulmonary hypertension

## Abstract

Exposure to hypoxia, due to high altitude or chronic lung disease, leads to structural changes in the pulmonary vascular wall, including hyperplasia and migration of pulmonary arterial smooth muscle cells (PASMCs). Previous studies showed that hypoxia upregulates the expression of Na^+^/H^+^ exchanger isoform 1 (NHE1) and that inhibition or loss of NHE1 prevents hypoxia-induced PASMC migration and proliferation. The exact mechanism by which NHE1 controls PASMC function has not been fully delineated. In fibroblasts, NHE1 has been shown to act as a membrane anchor for actin filaments, *via* binding of the adaptor protein, ezrin. Thus, in this study, we tested the role of ezrin and NHE1/actin interactions in controlling PASMC function. Using rat PASMCs exposed to *in vitro* hypoxia (4% O_2_, 24 h) we found that hypoxic exposure increased phosphorylation (activation) of ezrin, and promoted interactions between NHE1, phosphorylated ezrin and smooth muscle specific *α*-actin (SMA) as measured *via* immunoprecipitation and co-localization. Overexpression of wild-type human NHE1 in the absence of hypoxia was sufficient to induce PASMC migration and proliferation, whereas inhibiting ezrin phosphorylation with NSC668394 suppressed NHE1/SMA co-localization and migration in hypoxic PASMCs. Finally, overexpressing a version of human NHE1 in which amino acids were mutated to prevent NHE1/ezrin/SMA interactions was unable to increase PASMC migration and proliferation despite exhibiting normal Na^+^/H^+^ exchange activity. From these results, we conclude that hypoxic exposure increases ezrin phosphorylation in PASMCs, leading to enhanced ezrin/NHE1/SMA interaction. We further speculate that these interactions promote anchoring of the actin cytoskeleton to the membrane to facilitate the changes in cell movement and shape required for migration and proliferation.

## Introduction

Pulmonary hypertension (PH) is a complicated condition that can be fatal. Hypoxia is an inciting factor in some cases of PH, causing sustained contraction and thickening of the arterial wall. The structural changes in response to hypoxia include hyperplasia and increased migration of the pulmonary arterial smooth muscle cells (PASMCs), resulting in enhanced contractility and extension of smooth muscle into the precapillary arterioles. For patients with chronic lung disease, these hypoxia-induced structural and functional changes in the pulmonary vasculature correlate with the development of PH and increased mortality.

The exact mechanisms resulting in the hypoxia-induced hyperproliferative, hypermigratory PASMC phenotype are still being uncovered. Several studies (Quinn et al., 1998b; Rios et al., 2005; Yu et al., 2008; Yu and Hales, 2011; Walker et al., 2016) provided evidence that enhanced expression and/or activity of Na^+^/H^+^ exchanger isoform 1 (NHE1) is a contributing factor. NHE1 is a ubiquitous transmembrane exchanger that allows one Na^+^ ion into the cell in exchange for efflux of one H^+^ ion, regulating intracellular pH (pH_i_) in many cell types, including PASMCs (Quinn et al., 1991). Early studies demonstrated protective effects of inhibiting Na^+^/H^+^ exchange (NHE) on the development of pulmonary hypertension and vascular remodeling in response to CH (Quinn et al., 1998a; Quinn et al., 1998b). In later studies, we and others showed that mice lacking NHE1 were also protected from development of hypoxia-induced PH (Yu et al., 2008; Walker et al., 2016) and exhibited reduced vascular remodeling. We also showed that NHE1 protein expression is induced in PASMCs by chronic and subacute hypoxia (Rios et al., 2005; Shimoda et al., 2006; Walker et al., 2016), and studies, including from our lab, found that inhibiting NHE1 could reduce hypoxia-induced PASMC proliferation and migration (Yu and Hales, 2011; Walker et al., 2016).

The detailed mechanisms by which NHE1 controls PASMC function are still unclear. One study (Yu and Hales, 2011) suggested that NHE1 modulates proliferation through control of the transcription factor E2F1, whereas alteration in pH_i_ may also play a role as alkaline pH_i_ is associated with acute hypoxic vasoconstriction (Madden et al., 2001) and is required for PASMC proliferation (Quinn et al., 1996) while acidosis reduces pulmonary vascular remodeling (Christou et al., 2012). On the other hand, other studies suggested NHE1 can act as a membrane anchor or scaffold protein independent of its ion translocation properties (Denker et al., 2000; Denker and Barber, 2002; Putney et al., 2002). In fibroblasts and epithelial cells, NHE1 co-localized with and bound directly to ezrin (Denker et al., 2000; Baumgartner et al., 2004; Wu et al., 2004; Malo and Fliegel, 2006; Pedersen, 2006), a member of the ERM (ezrin/radixin/moesin) family of proteins. ERMs contain multiple binding domains and are important mediators of protein-protein interactions. Under basal conditions, the majority of ezrin exists in a folded, dormant state, with intramolecular binding between the N-terminal and C-terminal masking other protein-protein interaction domains (Reczek and Bretscher, 1998; Bretscher et al., 2002). Phosphorylation of ezrin by Rho kinase or PKC disrupts this intermolecular interaction (Matsui et al., 1998), allowing activated ezrin monomers to be available for binding to other protein targets, including NHE1 (Bretscher et al., 1997; Bretscher et al., 2000; Bretscher et al., 2002; Baumgartner et al., 2004). Indeed, only the phosphorylated form of the protein is found at the cell membrane (Hayashi et al., 1999). Of particular interest, the C-terminal of ezrin contains a binding site for filamentous actin, providing a possible link between NHE1 and cytoskeletal rearrangement, a process required for many cell functions, including contraction, hypertrophy, proliferation, and migration. Whether this scaffolding mechanism is operational in PASMCs is unclear, and the effect of hypoxia on phosphorylation of ezrin and potential NHE1/ezrin/actin interactions is unknown.

In this study, we examined whether increasing NHE1 was sufficient to induce changes in PASMC function in the absence of hypoxia, describe the effects of short-term hypoxic exposure on p-ezrin expression, determine whether NHE1, ezrin and actin form complexes within PASMCs, and test whether the effects of NHE1 on PASMC function may be due to its membrane anchoring, or scaffolding, properties *via* the ezrin binding site.

## Methods and materials

All protocols were reviewed by and performed in accordance with Johns Hopkins University Animal Care and Use Committee. Protocols and procedures comply with NIH and Johns Hopkins Guidelines for the care and use of laboratory animals.

### Exposure of mice to chronic hypoxia

Adult male C57B/6J mice (8–10 weeks, Jackson Labs) were randomly assigned to normoxia or chronic hypoxia. Mice were exposed to hypoxia in a chamber maintained at 10% O_2_ for 3 weeks. The chamber was constantly flushed with room air to maintain low (<0.5%) CO_2_ concentrations. A servo-control system (PRO-OX; Hudson RCI) monitored O_2_ levels and injected 100% N_2_ as needed to maintain 10% ± 0.5% O_2_. Cages were cleaned and food and water replenished twice per week. Normoxic animals were kept in room air on a wire rack adjacent to the chamber. All animals were allowed free access to food and water. At the end of exposure, left lungs were inflated *via* tracheal instillation of 0.5 mL 10% formalin, fixed and embedded in paraffin as previously described (Walker et al., 2016). Tissue sections (5 μm thick) were heated and washed with xylene to de-paraffinize sections, followed by graded ethanol washes to remove the xylene and rehydration (ethanol to water washes). Samples were subjected to heat-induced epitope (antigen) retrieval with a universal antigen retrieval kit (abcam), blocked and immunostained using a rabbit polyclonal antibody (abcam, ab47293) directed against phosphorylated ezrin (p-ezrin) with goat anti-rabbit Alexa 488 secondary antibody and counterstained with anti-mouse smooth muscle cell specific *α*-Actin (SMA) primary antibody (Sigma A2547) and goat anti-mouse CY3 secondary antibody. For each animal, 20 randomly selected small diameter pulmonary vessels, identified by location near bronchioles, architecture and luminal red blood cells, were selected using brightfield imaging. Once a vessel was identified, serial scans were obtained at 488 nm (Alexa 488) and 568 nm (CY3). Each vessel image was classified as positive (≥50% circumferential staining) or negative for SMA and p-ezrin.

### Isolation of pulmonary arterial smooth muscle cells

The methods for obtaining primary cultures of rat PASMCs have been previously described (Walker et al., 2016). Briefly, adult male Wistar rats (250–350 g) were deeply anesthetized with sodium pentobarbital (130 mg/kg i.p.) and the heart and lungs were removed *en bloc*. Because female rats are resistant to hypoxia, only male animals were used for this study. Intrapulmonary arteries (PAs; 200–600 µm o.d.) were dissected and cleaned of connective tissue in ice cold HEPES-buffered saline solution (HBSS) containing (in mM): 130 NaCl, 5 KCl, 1.2 MgCl_2_, 10 HEPES, and 10 glucose with pH adjusted to 7.2 with 5 M NaOH. The arteries were opened and the lumen gently scraped to remove endothelial cells. The arteries were allowed to recover for 30 min in cold (4°C) HBSS followed by 20 min in reduced-Ca^2+^ HBSS (20 μM CaCl_2_) at room temperature. The tissue was enzymatically digested for 20–25 min at 37°C in reduced-Ca^2+^ HBSS containing collagenase (type I; 1750 U/mL), papain (9.5 U/mL), bovine serum albumin (2 mg/mL) and dithiothreitol (1 mM). After digestion, single smooth muscle cells were dispersed by gentle trituration in Ca^2+^-free HBSS and were plated on 25 mm glass coverslips. PASMCs were cultured in Ham’s F-12 media containing 0.5% fetal calf serum and 1% penicillin/streptomycin for 24–48 h.

### *In vitro* hypoxic exposure

The methods for exposure of PASMCs to hypoxia have been previously reported (Shimoda et al., 2006; Walker et al., 2016). Briefly, culture plates containing cells at 80% confluence were placed in a modular chamber (Billups-Rothberg) gassed with 4% O_2_; 5% CO_2_. The chamber containing cells and the normoxic control plates were placed in a room air incubator maintained at 37°C and 5% CO_2_. Oxygen levels inside the hypoxic chamber were monitored using a hand-held oxygen monitor (model 5577; Hudson RCI).

### Immunoblot

Total protein was extracted from PAs and PASMCs in ice-cold T-PER buffer containing protease inhibitors (Roche Diagnostics). Quantification of proteins was performed using BCA protein assay (Pierce) and equal amounts of total protein per lane were separated by electrophoresis through a 10% SDS-PAGE gel and transferred onto polyvinylidene difluoride membranes. Membranes were incubated in 5% non-fat dry milk in Tris-buffered saline containing 0.2% Tween 20 to block non-specific binding sites before being probed with primary antibody against NHE1 (United States Biological, 146226), HA (Cell signaling 6e2 #2367S) or p-ezrin (abcam, ab47293). Bound antibody was probed with horseradish peroxidase-conjugated anti-rabbit or anti-mouse secondary antibody (1:10,000; Sigma) and detected by enhanced chemiluminescence. Membranes were then stripped and re-probed for *β*-Tubulin (1:10,000, Sigma) as a housekeeping protein. Protein levels were quantified by densitometry using ImageJ.

### Immunofluorescence

PASMCs were plated and grown on glass slides until desired confluency, usually 70%–80%. Cells were treated with ezrin phosphorylation inhibitor NSC668394 (0.1 or 0.5 µM; Millipore) or vehicle (DMSO) and placed in the hypoxic chamber at 4% O_2_ for 24 h. Cells were then washed, fixed with 4% formalin, and permeabilized with acetone. PASMCs were blocked and incubated with antibodies against SMA (Sigma, A2547, 1:500) and NHE1 (Thermo Fisher, PA5 115917 1:100) followed by incubation with fluorescent-conjugated secondary antibody (Cy5 goat anti-mouse for SMA and FITC goat anti-rabbit for NHE1, Life Technologies, Carlsbad, CA, United States) and DAPI nuclear counterstain. Images were obtained using a microscope with fluorescence objectives (Olympus I × 51, Center Valley, PA, United States) by an investigator blinded to treatment groups.

### FRET

Cells were formalin fixed, permeabilized and stained with rabbit anti-NHE1 antibody (United States Biologicals, N2015-06.100) conjugated to goat anti-rabbit Alexa 488 (green), mouse anti-SMA (Sigma, A2547) conjugated to goat anti-mouse CY3 (red). In some experiments, the nuclear stain YO-PRO (green) was added to help visualize cells. FRET efficiency was performed using confocal microscopy and standard protocols. Cells were excited at 488 nM and emitted fluorescence for each fluorescent stain was measured, followed by photobleaching (30 scans at 100% lamp intensity) of the acceptor dye (CY3) and re-measuring fluorescence at each wavelength. If FRET was initially present, a resultant increase in donor fluorescence will occur upon photobleaching of the acceptor. The energy transfer efficiency was quantified as: *FRET eff* = (Dpost-Dpre)/Dpost, where Dpost is the fluorescence intensity of the donor (Alexa 488) after acceptor photobleaching, and Dpre the fluorescence intensity of the donor before acceptor photobleaching. An efficiency heatmap was generated for each cell. Following acquisition of the FRET images, an investigator blinded to treatment groups used the SMA image to select 10 regions of interest per cell that corresponded with SMA filaments. For each region of interest, FRET efficiency was calculated as above, and averaged to get a single value per cell.

### Co-immunoprecipitation

Samples were collected into solubilization buffer, lysed and cell debris pelleted by centrifugation. Total protein content in the input (total cell lysate) was assessed *via* BCA protein assay and volumes adjusted to normalize protein content in each sample. Samples were precleared by incubating with IgG and protein A+ beads and then incubated with either IgG (Control) or antibody directed against SMA (Sigma, A2547), p-ezrin (abcam, ab47293), NHE1 (Millipore, AB3081) or HA (Cell signaling 6e2 #2367S). Protein A+ beads were added to each sample, incubated and pelleted. Beads were washed and bound proteins eluted. Beads were removed *via* centrifugation and the proteins in an equal volume of supernatant and eluent for each sample were separated *via* electrophoresis and transferred to PVDF membranes. Membranes were probed with antibodies directed against the proteins of interest.

### Generation of adenoviral constructs

A cDNA for human NHE1 cloned into pCMV-Sport6 was obtained from the MGC Collection (Genbank Accession # BC051431) through Open Biosystems, Inc. (Birmingham, ALA). To reduce unintentional nucleotide changes caused by the PCR errors, a small fragment of the total cDNA encoding nucleotides 1638–2582 was generated by PCR using primers that also added an epitope tag (2 x HA) to the C-terminus of the protein. Following verification of the DNA sequence, to remove extraneous 3′ untranslated region and 5′ UTR sequences, the remainder of the coding sequence (nucleotides 120–1637) was replaced by a PCR product into the KpnI/SalI sites of pCMVSport 6. These maneuvers resulted in a full-length cDNA. Because pCMVSport6 contains attB1/attB2 recombination sites compatible with the GATEWAY recombination cloning system, NHE1 cDNA was transferred by recombination cloning first into pDONR221 and then into pAdDEST-V5 to generate the plasmid, pAdCMV-NHE1-2XHA (AdNHE1) using manufacturer’s instructions (InVitrogen). Replication defective virus was prepared following transfection of the plasmids into 293A cells and isolation of cell extracts showing cytopathic effects. This cell extract was then used to amplify the virus. Viral titer was determined using the Clontech Viral Titer kit and virus purification was performed using Adenopure Filter system (PureSyn, Inc.). Site directed mutagenesis was performed to create a NHE1 construct the was unable to bind to phosphorylated ezrin (AdMut) as previously described (Denker et al., 2000).

### Adenovirus infection of RPASMCs

Rat PASMCs were plated in culture dishes in Ham’s F-12 media supplemented with 10% FCS and penicillin/streptomycin until 80% confluent. Cells were then placed into Ham’s F-12 basal media supplemented with 0.5% FCS and penicillin/streptomycin 24 h prior to infection. PASMCs were infected with 50 MOI of a control adenovirus containing GFP (AdGFP), AdNHE1 (wild-type), or AdMut (NHE1 with a mutation in the ezrin binding) for 4 h at 37°C, after which dishes were washed with phosphate buffered saline, and placed into basal media for an additional 24 h at 37°C before cells were trypsinized and plated for migration, proliferation, and pH experiments.

### pH_i_ measurements

pH_i_ within PASMCs was monitored using the cell permeant pH sensitive dye 2′,7′-bis(carboxyethyl)-5(6)-carboxyflourescein (BCECF AM) as previously described (Rios et al., 2005; Shimoda et al., 2006). Cells were placed in the perfusion chamber, perfused with modified Krebs-Ringer Bicarbonate solution and excited with light filtered at 490 and 440 nm. Light emitted from the cells was detected at 530 nm. The ratio of 490 to 440 nm emission was calculated and pH_i_ was estimated from *in situ* calibration after each experiment. For calibration, PASMCs were perfused with a high K^+^ solution containing (in mM): 105 KCl, 1 MgCl_2_, 1.5 CaCl_2_, 10 glucose, 20 HEPES-Tris, and 0.01 nigericin to allow pH_i_ to equilibrate to external pH (42). A two-point calibration was created from fluorescence measured as pH_i_ was adjusted with KOH from 6.5 to 7.5. Intracellular H^+^ ion concentration ([H^+^]_i_) was determined from pH_i_ using the formula: pH_i_ = -log ([H^+^]_i_). Cells were perfused using a multi-input, single output system connected to reservoirs containing control solutions or solutions containing various compositions. Switching between reservoirs allowed for rapid exchange of the chamber solution. NHE activity was measured as previously described (Rios et al., 2005).

### Cell migration

Equal numbers of cells were seeded onto 24 mm polystyrene transwell inserts with transparent, permeable (8 µM pore) filters. Across different experiments, the number of cells initially plated ranged between 20,000–50,000; however, within any given experiment (i.e., hypoxic exposure, overexpression, etc.) a fixed number of cells was used across groups. Cells were placed in 3 mL of Ham’s F-12 media supplemented with 0.5% FCS and 1% penicillin/streptomycin and allowed to adhere for 1–2 h before addition of drugs or beginning hypoxic exposures. In some experiments, cells were treated with 0.5 µM ezrin phosphorylation inhibitor NSC668394 (Millipore) or vehicle (DMSO) for 1 h prior to hypoxic exposures. After 24 h, filters were washed with PBS, and cells fixed in ice cold 95% EtOH for 10 min. Following fixation, cells were washed with PBS and stained with Coomasie blue for 5 min at room temperature. Filters were washed and placed in PBS. Each filter was examined under ×40 magnification and images of at least five fields per filter were obtained at random to determine the total number of cells. The site of each field was marked on the bottom of the culture dish. The tops of the filters were then scraped with a cotton swab being careful not to move them within the culture dish, rinsed with PBS to remove non-adherent cells, and images of the same fields were again obtained (migrated cells). Once images of all fields were obtained, the number of cells in each image (total and migrated) was counted. Cell migration was expressed as the number of migrated cells normalized to the number of total cells (% total cells).

### Cell proliferation

Equal numbers of cells (5000/well) were placed in the wells of a 96-well plate, with each condition repeated in at least duplicate. Ham’s F-12 media containing 0.5% FCS and 1% penicillin/streptomycin was added to each well for a total volume of 200 µL. Cells were incubated for 1 hour before treatments (NSC668394 or vehicle) were added. Cells were incubated at 37°C under control (21% O_2_; 5% CO_2_) or hypoxic (4% O_2_; 5% CO_2_) conditions for 72 h before addition of 20 µL of labeling BrdU. Cells were allowed to incubate for additional 24 h in the presence of BrdU. Incorporation of BrdU into proliferating cells was measured using an ELISA (Cell Biotrak, Sigma) according to the manufacturer’s instructions. After 10–30 min of color development, reactions were stopped and well values were measured at 450 nm with an ELISA plate reader. Each treatment was run in multiple wells and the values were averaged. Because development times could vary between experiments, samples were normalized to the control sample for that experiment.

### Data Analysis

Data are expressed as scatter plots with bars representing means ± SEM. Each dot represents a separate experimental run, and since all experimental runs were performed on tissue/cells from different animals, “n” also refers to the number of animals. For pH_i_ experiments, data was collected from 10–30 cells per coverslip, and averaged to obtain a single value for each biological replicate per experiment. All data were tested for normality prior to running statistical tests and in some cases, data were transformed using arctan transformation to create a normal distribution. Statistical comparisons were performed using Student’s *t*-test for data in two groups, or one- or two-way ANOVA with a Holm-Sidak *post hoc* test for multiple group comparisons. All immunofluorescence images within an experiment were captured on the same day using the same imaging conditions/setting. For all imaging experiments (immunofluorescence, FRET, migration), the investigator obtaining the images and performing counting was blinded to treatments/groups.

## Results

### Effect of increasing NHE1 levels on PASMC function

**FIGURE 1:**
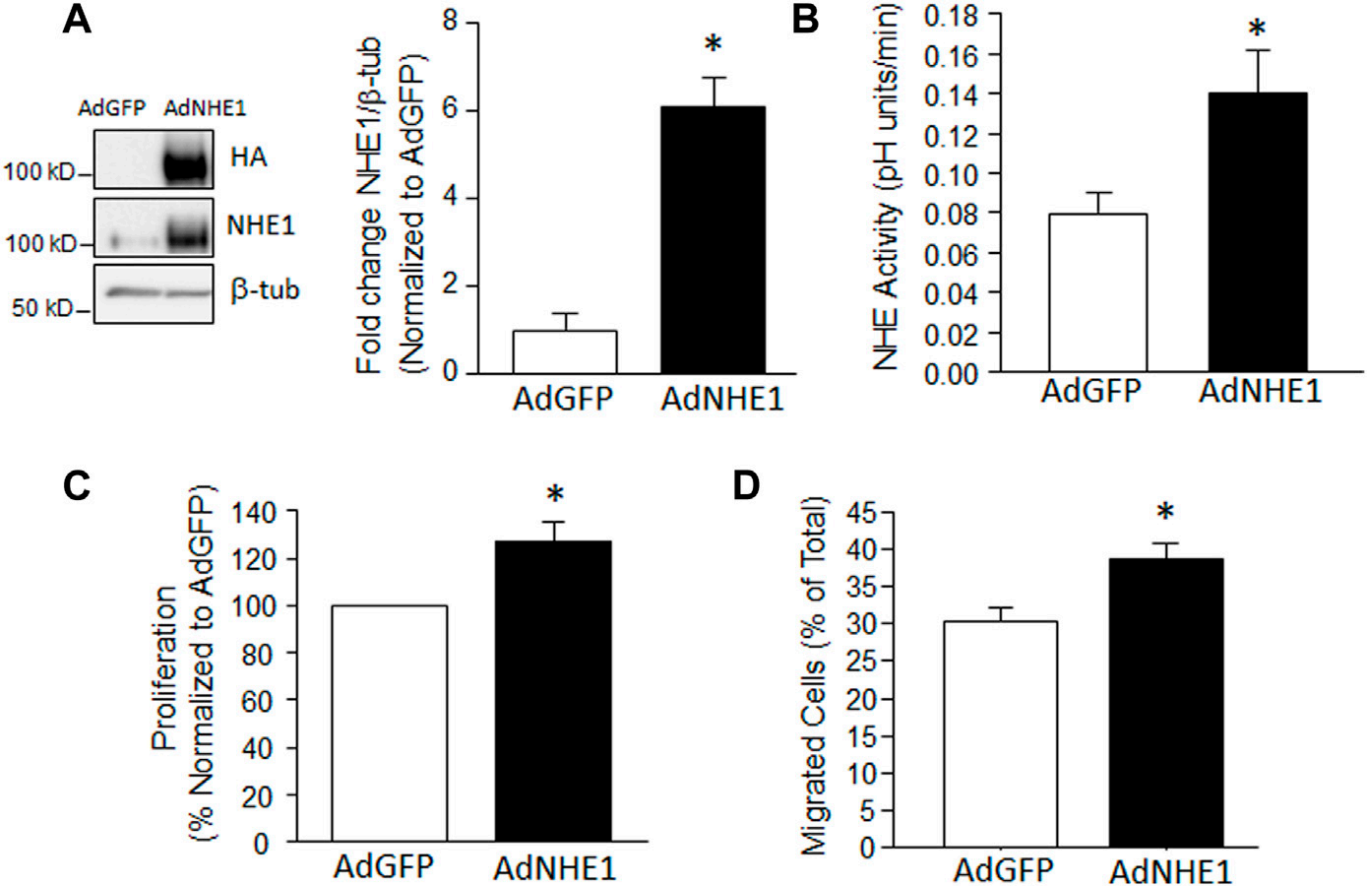
Increased NHE1 levels and cell function. **(A)** lmmunoblot showing overexpression of NHE1 protein achieved using an adenoviral construct containing wild-type NHE1 (AdNHE1) tagged with hemagglutinin (HA). Top panel shows expressed protein, while middle panel shows total NHE1 (expressed plus native) in cells infected with AdNHE1 or AdGFP (control). β-Tubulin (β-tub) was used as a loading control. Bar graph shows mean ± SEM values for total NHE1 protein (*n* = 4 each). **(B–D)** Bar graphs show mean ± SEM for **(B)** Na^+^/H^+^ exchange (NHE) activity (*n* = 7 each); **(C)** proliferation (*n* = 6 each), and **(D)** migration (*n* = 5 each) in PASMCs infected with AdGFP and AdNHE1. * indicates significant difference (*p* < 0.05) compared to AdGFP.

While we previously demonstrated that inhibiting NHE1 prevents hypoxia-induced increases in migration and proliferation in PASMCs, it was unknown whether increasing NHE1 levels was sufficient to mimic the effects of hypoxia on cell function. Thus, we infected PASMCs with adenoviral constructs containing either HA-tagged wild-type NHE1 (AdNHE1) or GFP (AdGFP) as a control. We were able to demonstrate significant upregulation of NHE1 protein expression (Figure 1A) using AdNHE1. The increase in expression was accompanied by an enhancement of NHE activity (Figure 1B), suggesting that our expressed protein was trafficking properly to the membrane and functioning correctly. In PASMCs where we artificially enhanced NHE1 expression, both proliferation (Figure 1C) and the percent of migrating cells (Figure 1D) were significantly increased compared to cells infected with AdGFP. These results suggest increasing NHE1 was sufficient to induce cell migration and proliferation in the absence of hypoxia.

### NHE1-actin interactions in PASMCs

**FIGURE 2:**
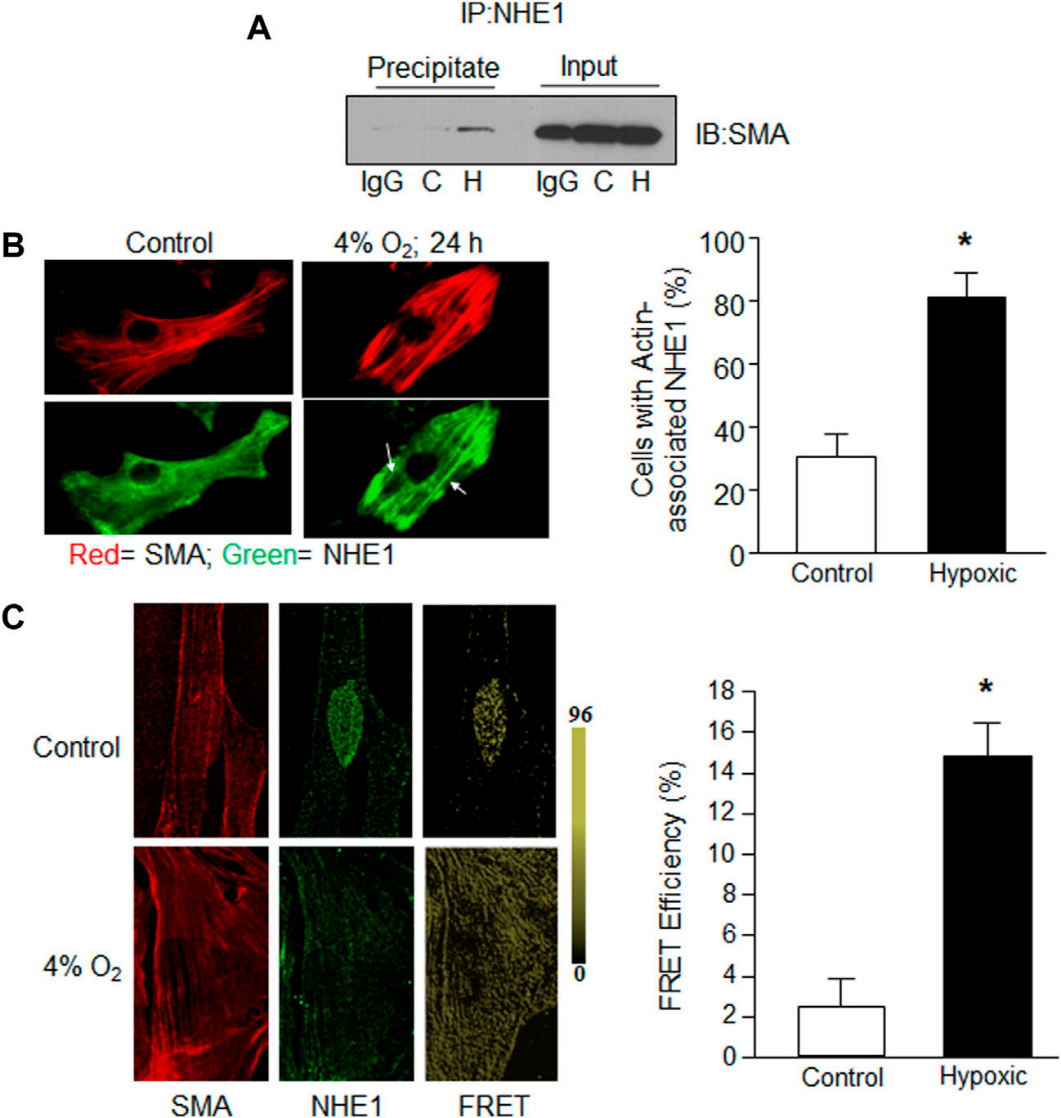
Evidence for NHE1/actin interactions in hypoxic PASMCs. Rat PASMCs were exposed to control (C; 21% O_2_) or hypoxic (H; 4% O_2_) conditions for 24 h. **(A)** Proteins obtained following immunoprecipitation using anti-NHE1 were probed for expression of smooth muscle specific α—actin (SMA). In the first lane for both precipitate and input (total cell lysate; control), IgG was used in place of anti-NHE1 as a control. A similar increase in SMA/NHE1 interaction with hypoxia was observed in a second experiment. **(B)** Immunofluorescence images showing NHE1 (green) and SMA (red) expression in control (left) and hypoxic (right) PASMCs. Bar graph shows mean ± SEM for percent of cells exhibiting NHE1 that associated with actin filaments (*n* = 6 each). **(C)** Confocal images showing individual SMA (left) and NHElimages (middle) for a control cell prior to photobleaching, and FRET values after acceptor photobleaching (right) for a control (top) and hypoxic (bottom) PASMC. Bar graphs show mean ± SEM FRET efficiency between control and hypoxic PASMCs (*n* = 10 cells from three separate experiments for each condition). * indicates significant difference (*p* < 0.05) compared to control.

Given the variable effects of short-term hypoxia on NHE1 expression we previously observed (Walker et al., 2016), we next explored whether short-term hypoxia might change the localization or protein-protein interactions of NHE1. In particular, we looked to reports showing that NHE1 binds with the phosphorylated form of the adaptor protein, ezrin (p-ezrin), to mediate NHE1/actin filament interactions in fibroblasts (Denker et al., 2000). We used co-immunoprecipitation to investigate the binding of NHE1 and actin in PASMCs, and whether this interaction was altered by short-term hypoxia. Initial experiments indicated that in PASMCs maintained under control conditions, little SMA co-immunoprecipitated with NHE1, whereas in PASMCs exposed to hypoxia (4% O_2_; 24 h) a band for SMA was clearly visible in the precipitated fraction (Figure 2A). No band was observed in control samples incubated with IgG indicating that non-specific binding was minimal.

We next examined NHE1 localization using immunofluorescence. In PASMCs maintained under control conditions, actin filaments could be observed while NHE1 typically exhibited a diffuse pattern of staining (Figure 2B). In contrast, in cells exposed to hypoxia, NHE1 staining exhibited a similar pattern as SMA, appearing to align with the actin filaments, reflected in an increase in the percent of cells with SMA-associated NHE1 localization.

In addition to co-immunoprecipitation and conventional immunofluorescence methods, we confirmed NHE1/actin interactions in rat PASMCs exposed to control conditions or hypoxia (4% O_2_, 24 h) using fluorescence resonance energy transfer (FRET) with acceptor bleaching, or dequenching. Normoxic PASMCs showed only sparsely localized FRET, whereas in PASMCs exposed to hypoxia, FRET was observed throughout the cell (Figure 2C). These results confirm that NHE1 and SMA form complexes in PASMCs and that the level of interaction is increased by hypoxia.

### Effect of hypoxia on ezrin expression and interaction with actin

**FIGURE 3:**
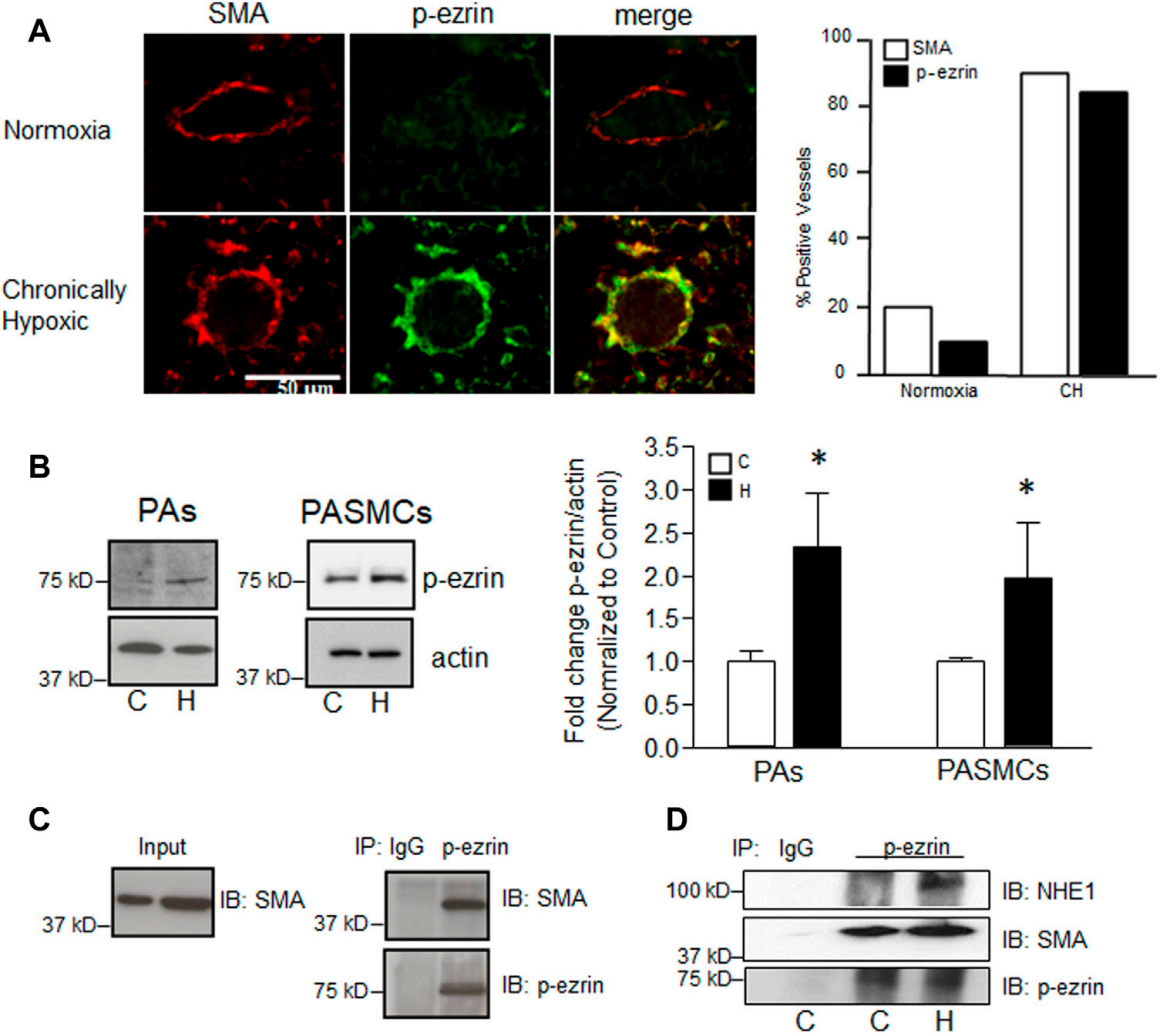
Effect of hypoxia on phosphorylation of ezrin. **(A)** Representative confocal images showing enhanced immunofluorescence for phosphorylated ezrin (p-ezrin; Alexa 488) in smooth muscle cells (indicated by positive staining for smooth muscle specific α-actin; SMA; Cy3) in a small pulmonary vessel in mice exposed to normoxia (top) or chronic hypoxia (CH; 10% O_2_, 3 weeks; bottom). Bar graph represents mean number of vessels (out of 20 randomly selected) staining positive for p-ezrin and SMA. Data from 1 normoxic and 1 CH lung. **(B)** Immunoblot shows protein levels for p-ezrin in pulmonary arteries (PA) from normoxic and chronically hypoxic rats and in PASMCs from control rats exposed to control (C) or hypoxic (H; 4% O_2_; 24 h) conditions. Bar graph shows mean ± SEM for fold change (compared to C) in p-ezrin protein levels (normalized to β-actin) in control and hypoxic PA (*n* = 3 each) and PASMCs (*n* = 5 each). **(C)** Immunoblot demonstrating p-ezrin co-immunoprecipitates with SMA. Similar results were obtained in four separate experiments. **(D)** Immunoblot shows enhanced NHE1 and SMA immunoprecipitation with p-ezrin in hypoxic (H) compared to control (C) PASMCs. IgG was used as a control. Similar results were obtained in two other experiments. * indicates significant difference (*p* < 0.05) compared to control.

Previous reports in fibroblasts indicated that NHE1/actin filament interactions were mediated by the adaptor protein, ezrin (Denker et al., 2000). Ezrin is typically found in an inactive state in the cytosol, with phosphorylation at Thr567 required for activation and binding to actin. To determine the level of ezrin activation in PASMCs, and the effect of hypoxia, we used several approaches. To understand the potential role of p-ezrin in vascular remodeling in chronically hypoxic animals, we initially performed preliminary experiments to determine whether upregulation/activation of ezrin occurred *in vivo* using fixed tissue from normoxic and chronically hypoxic mice (Figure 3A). Using lung tissue from 1 normoxic and 1 chronically hypoxic mouse, we found that p-ezrin levels were enhanced in smooth muscle cells (SMA positive) in small diameter pulmonary vessels, where the majority of remodeling occurs, as indicated by the increased appearance of SMA (Walker et al., 2016; Abud et al., 2012). We corroborated these initial findings in endothelial-denuded small diameter pulmonary arteries from normoxic and chronically hypoxic rats (Figure 3B) and in PASMCs exposed to hypoxia *in vitro* (4% O_2_; 24 h). In both cases, limited p-ezrin protein was detected in control PAs or PASMCs. We found p-ezrin levels were higher in hypoxic PA and PASMC samples compared to normoxic samples.

Our next step was to determine whether p-ezrin interacted with SMA in our cell type. Using co-immunoprecipitation, we found that SMA readily precipitated with p-ezrin (Figure 3C). When comparing PASMCs maintained under control conditions and those exposed to hypoxia (4% O_2_; 24 h) we found that little NHE1 precipitated with p-ezrin in control cells, whereas a band was readily observed in hypoxic PASMCs (Figure 3D). In contrast, in the same samples, SMA precipitated with p-ezrin in both control and hypoxic conditions.

### Validating the NHE1 ezrin-binding mutant

**FIGURE 4:**
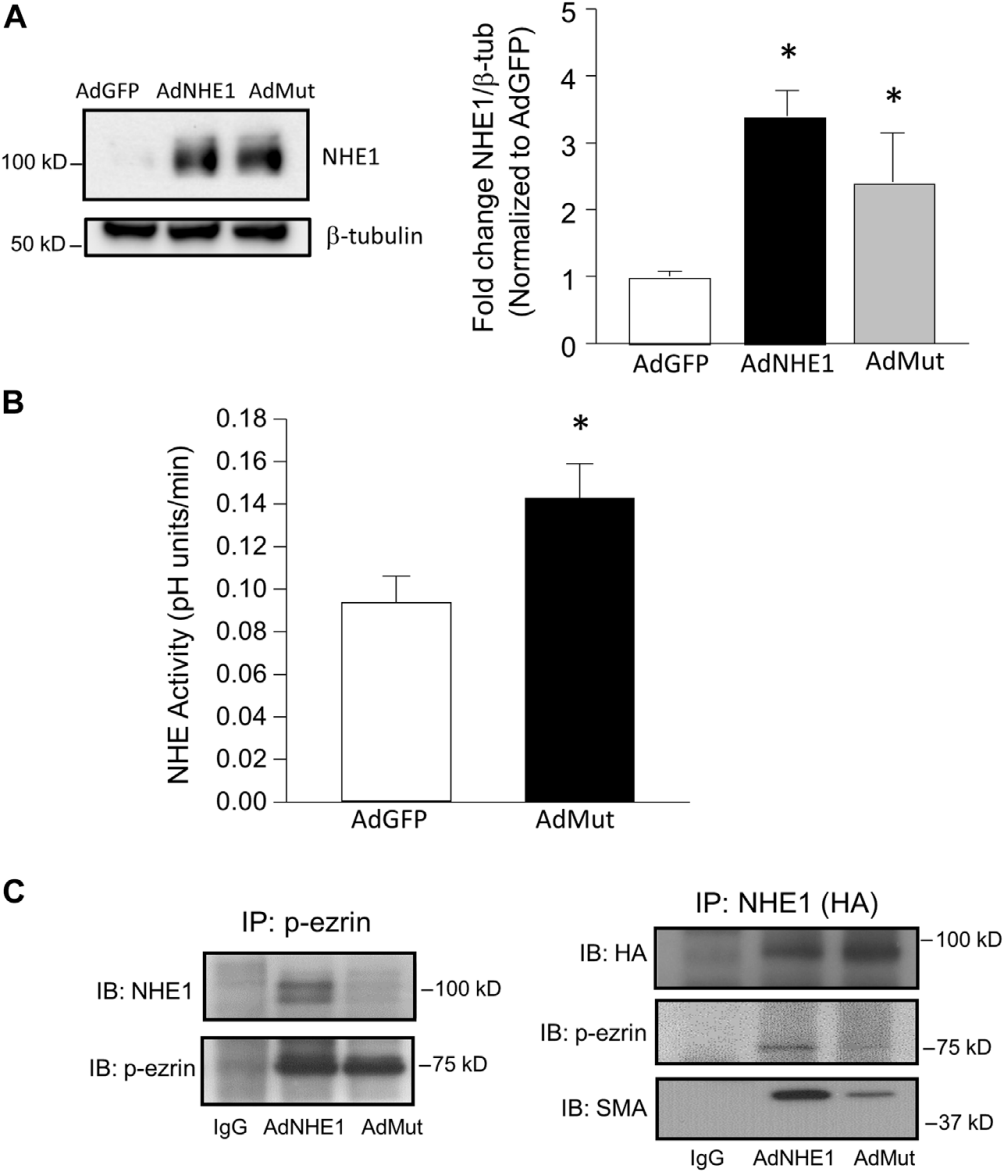
Validation of NHE1 ezrin binding mutant (AdMut). **(A)** Protein levels of expressed NHE1 mutant (AdMut) compared to wild-type NHE1 (ANHE1) in cells infected with AdGFP (control). Bar graph shows mean ± SEM NHE1 protein expression (normalized to β-tubulin) in PASMCs infected with AdGFP, AdWT, and AdMut (*n* = 3 each). **(B)** Bar graph shows mean ± SEM Na^+^ /H^+^ exchange (NHE1) activity in PASMCs infected with AdGFP or AdMut (*n* = 6 each). * indicates *p* < 0.05 compared to AdGFP. **(C)** Immunobtots show co-immunoprecipitation of p-ezrin with WT but not mutant NHE1. Left blots show AdWT but not AdMut immunoprecipitated with p-ezrin. Right blots show loss of p-ezrin precipitation with AdMut. In this experiment, SMA precipitation with mutant NHE1 (AdMut) was also reduced. IgG precipitation in cells expressing AdWT was used as a control. SMA and p-ezrin interactions with WT/mutant NHE were assessed in three separate experiments.

To test whether ezrin/NHE1 interactions were involved in the functional response to hypoxia, we created a HA-tagged form of NHE1 where the ezrin binding site was mutated based on previously reported information (Denker et al., 2000). Using adenoviral constructs containing this mutant (AdMut), we tested the expression levels of the mutated protein in our cells (Figure 4A). While only a faint band for NHE1 was detected in AdGFP-infected cells, PASMCs infected with either wild-type NHE1 (AdNHE1) or the ezrin building mutant (AdMut) had significant NHE1 protein expression. The ezrin binding mutant appeared to localize properly to the membrane and exhibit intact NHE exchange activity (Figure 4B), as NHE activity measured in PASMCs with the ezrin-binding mutant had similar elevated levels of NHE activity as PASMCs infected with wild-type NHE1 (Figure 1B). To test whether our mutant NHE1 was still capable of interacting with ezrin, we performed co-immunoprecipitation using HA-tagged NHE1 as the bait. Use of IgG resulted in no detectable bands for p-ezrin in the precipitate (Figure 4C). In PASMCs expressing wild-type NHE1, a band for p-ezrin was detected in the precipitate, whereas the amount of p-ezrin in the precipitate from PASMCs infected with AdMut was barely visible. When these precipitates were probed for SMA, a strong band was detected in PASMCs infected with AdNHE1, with reduced SMA detected in PASMCs infected with AdMut. We also confirmed our co-immunoprecipitation experiments by performing the reverse pull-down, using p-ezrin. In this case, HA-tagged NHE1 protein was only detected in samples from PASMCs infected with wildtype NHE1, and not in cells infected with AdMut, AdGFP or when IgG was used for immunoprecipitation. In contrast, similar bands for SMA were observed under all conditions. These results confirmed loss of NHE1/ezrin interactions with our mutant.

### Role of NHE1/ezrin interactions in cell function

**FIGURE 5:**
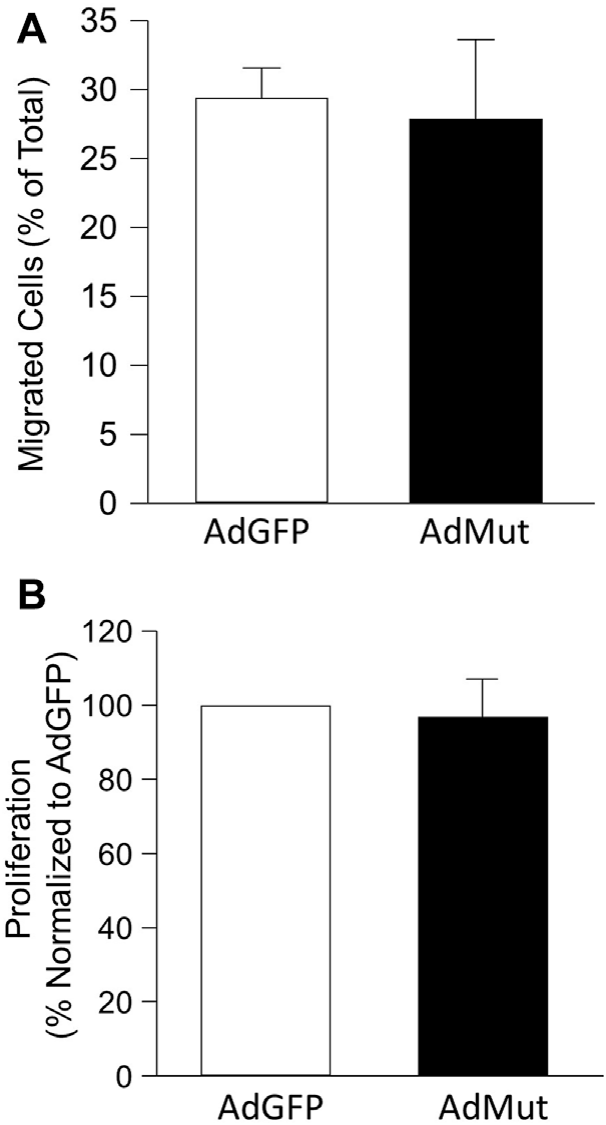
Effect of NHE1 with ezrin binding mutation on PASMC function. Bar graphs represent mean ± SEM values for **(A)** migration (*n* = 3 each) and **(B)** proliferation (*n* = 5 each) in PASMCs infected with AdGFP (control) or NHE1 that is unable to bind ezrin (AdMut).

To test the role of ezrin/NHE1 interactions in NHE1-induced changes in PASMC function, we again infected cells with AdGFP or AdMut and measured migration and proliferation. While forced expression of wild-type NHE1 increased both migration and proliferation in PASMCs (Figure 1), expressing the mutant NHE1 had no significant effect on cell migration or proliferation (Figure 5) when compared to cells infected with AdGFP.

### Effect of inhibiting ezrin phosphorylation in PASMCs

**FIGURE 6:**
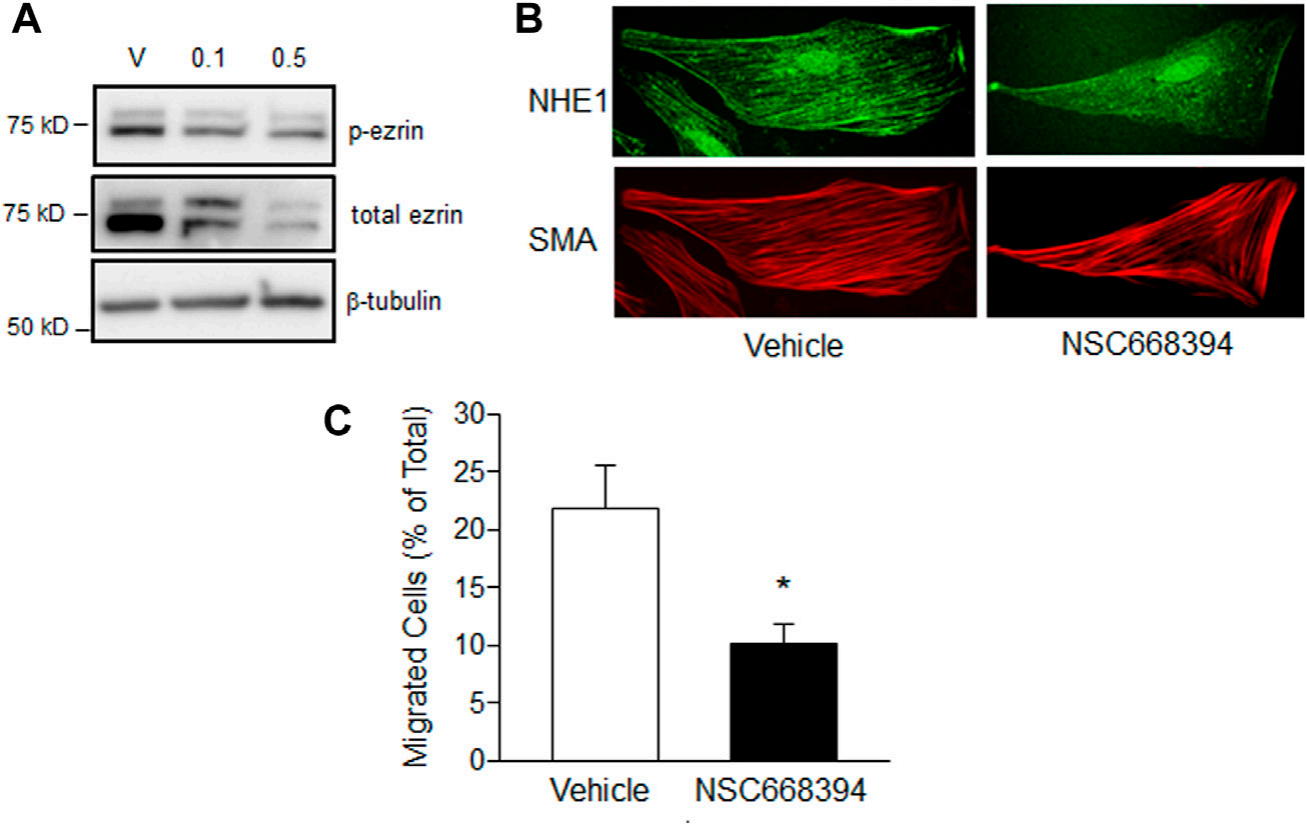
The effect of ezrin inhibition on PASMC function. **(A)** Immunoblot showing effect of treatment with vehicle (DMSO) or the ezrin phosphorylation inhibitor, NSC668394 (0.1 or 0.5 μM), for 72 h on ezrin phosphorylation (p-ezrin). Similar results were obtained in two separate experiments. **(B)** Immunofluorescence images showing NHE1 and smooth muscle specific α-actin (SMA) co-localization in hypoxic PASMCs treated with vehicle (DMSO) or NSC668394 (0.1 μM 24 h). **(C)** Bar graphs showing mean ± SEM values for migration in PASMCs treated with vehicle or 0.5 μM. NSC668394 and exposed to hypoxia (4% O_2_; 24 h; *n* = 7 each). * indicates *p* < 0.05 compared to vehicle.

Finally, we tested the effect of inhibiting ezrin phosphorylation, and by extension, binding to NHE1 and actin, on cell function during hypoxia. Our initial tests using NSC668394, a cell permeable inhibitor that binds to ezrin and prevents phosphorylation at The567, thereby inhibiting activation of ezrin, at concentrations shown to be effective in other cell types (Bulut et al., 2012; Pore et al., 2015) resulted in widespread PASMC death (data not shown). Using lower concentrations, we were able to demonstrate reduced ezrin phosphorylation up to 72 h (Figure 6A). We next treated PASMCs with NSC668394 (0.1 μM) or vehicle (equal volume DMSO) and exposed them to hypoxic conditions (4% O_2_; 24 h) and assessed NHE1/SMA co-localization (Figure 6B). NHE1/SMA co-localization was readily apparent in vehicle-treated PASMCs exposed to hypoxia (90% of cells with SMA-associated NHE1), whereas limited co-localization was observed in hypoxic PASMCs treated with NSC668394 (34.6% of cells with SMA-associated NHE1). Finally, we measured the effect of p-ezrin inhibition on cell function. While 0.1 μM NSC668304 largely repressed NHE1/SMA co-localization, this concentration had variable effects on migration (data not shown), so we measured migration with the higher concentration of the inhibitor. We found NCS668304 reduced migration in hypoxic PASMCs (Figure 6C). We also noticed a decrease in cell adherence with NSC668394 (−29.7% ± 10.6%; *n* = 3), even under control (i.e., non-stressed) conditions. Because our proliferation assay requires equal numbers of adherent cells, we did not pursue experiments to assess the effect of the inhibitor on PASMC proliferation.

## Discussion

In this study, we describe the role of NHE1/p-ezrin interactions in mediating NHE1-dependent changes in PASMCs function. Under hypoxic conditions, NHE1 localization changes to align with SMA fibers. Hypoxia-induced increases in p-ezrin levels are associated with enhanced NHE1/SMA interactions, which we show are required to facilitate PASMC migration and proliferation using a form of NHE1 that is unable to bind p-ezrin. Finally, we show that reducing the level of p-ezrin using a pharmacological inhibitor attenuates hypoxia-induced migration.

In previous studies, we demonstrated an upregulation of NHE1 mRNA and protein expression during prolonged hypoxic exposures (Rios et al., 2005; Shimoda et al., 2006). Moreover, pharmacological inhibition of NHE activity or loss of NHE1 protein by either silencing or genetic deficiency prevents both hypoxia-induced pulmonary arterial smooth muscle cell migration and proliferation as well as pulmonary vascular remodeling in chronically hypoxic mice and rats (Quinn et al., 1998a; Quinn et al., 1998b; Yu et al., 2008; Yu and Hales, 2011; Walker et al., 2016). While these studies clearly indicate a role for NHE1 during hypoxia, we wanted to determine whether increasing NHE1 expression and activity was sufficient to induce changes in PASMC function. Using an adenoviral approach to increase expression of NHE1 in PASMCs revealed that PASMC migration and proliferation could be enhanced by increasing NHE1 expression, indicating that other factors associated with hypoxia are not required to initiate the response.

We previously determined that hypoxia-induced proliferation of PASMCs requires several days to be reliably measured, perhaps relating to the time required for hypoxia to increase total NHE1 protein levels (Rios et al., 2005; Shimoda et al., 2006). Interestingly, changes in PASMC migration can be observed with short-term (i.e., 24 h) exposure to moderate hypoxia (Walker et al., 2016); however, at this time point, changes in NHE1 mRNA and protein are not readily apparent (Walker et al., 2016). One possibility is that upregulation of NHE1 at 24 h is variable and difficult to detect *via* immunoblot, but that by 48 h sufficient accumulation of protein allows for measurement. Thus, small increases in NHE1 might be able to drive the change in phenotype. Another possibility is that while increasing NHE1 protein levels is sufficient to induce changes in cell function, there may be other mechanisms that contribute to these effects prior to an increase in NHE1 protein levels. One such potential mechanism could be interactions between NHE1 and the adaptor protein, ezrin. Once phosphorylated, active ezrin can form a linkage between the cytosolic C-terminal tail of NHE1 and actin filaments in fibroblasts and epithelial cells (Denker et al., 2000; Denker and Barber, 2002). To test whether a similar complex exists in PASMCs, we used several methods to assess NHE1/ezrin/SMA interactions, including co-immunoprecipitation, co-localization assessed *via* immunofluorescence and FRET. Using these multiple methods, we demonstrated interactions between NHE1 and SMA that increase during hypoxia, resulting in NHE1 protein appearing to align along actin fibers. In this case, NHE1 may act as a membrane anchor for the cytoskeleton. We also showed that phosphorylated, or active, ezrin forms complexes with both NHE1 and SMA. We then tested whether these interactions contribute to changes in cell function by mutating the ezrin binding site in the C-terminal of NHE1 as previous described by others (Denker et al., 2000; Denker and Barber, 2002). NHE1 lacking the p-ezrin binding site could still traffic properly and localize in the membrane, as demonstrated by an increase in NHE activity in cells expressing the NHE1 mutant similar to that measured in cells where wild-type NHE1 was overexpressed. Loss of NHE1/p-ezrin binding in the mutant was confirmed by co-immunoprecipitation. Of note, although the ability of mutant NHE1 to precipitate with p-ezrin and SMA was reduced, p-ezrin could still interact with SMA, showing specificity for the interaction (i.e., mutating NHE1 did not disrupt p-ezrin/SMA binding). The fact that a small amount of p-ezrin and SMA could still be found in the eluent when mutant NHE1 was used as a precipitate may reflect the fact that NHE1 forms dimers. Since the infected cells also expressed native NHE1, albeit to a much lower level, it is a strong possibility that some NHE1 mutant proteins may have complexed with p-ezrin and SMA through formation of native/mutant heterodimers. Non-etheless, the NHE1 mutant was still incapable of inducing migration or proliferation of PASMCs, suggesting a critical role for the ezrin binding site in controlling cell function.

We also tested the ability of an ezrin inhibitor to prevent hypoxia-induced changes in cell function. To our surprise, we found that use of NSC668394 at published concentrations (1–10 μM) caused significant cell death and/or detachment in PASMCs. The unexpected death/detachment may be a more prominent feature of primary cells, as most published reports used the drug in cell lines often originating from tumors (Bulut et al., 2012; Pore et al., 2015). At concentrations of 0.1 and 0.5 μM, we still detected a decrease in p-ezrin levels. Interestingly, total ezrin levels were also reduced after treatment with the inhibitor, which was unexpected. In looking at the literature, most other studies with short duration treatment (1–6 h; Bulut et al., 2012; Proudfit et al., 2020) have not observed a reduction in total ezrin levels, although one study did observe results consistent with ours (Ghaffari et al., 2019). These results may suggest that in certain tissues or with longer time points, as used in this study, negative feedback may act to reduce ezrin protein levels in addition to phosphorylation. We chose these longer time points to confirm continued inhibition at time points consistent with when we were performing our assays. The reduction in total and p-ezrin was associated with lower NHE1/actin co-localization and a significant reduction in migration under hypoxic conditions, despite the fact that we did not fully abolish ezrin phosphorylation at the concentrations used in our experiments. We also noted a decrease in cell adherence (i.e., total cell count) in our migration experiments, suggesting either a continued level of cell death at lower concentrations or that the cells detached from the membrane when inhibitor was added. Because the actin cytoskeleton plays a prominent role in mediating changes in cell shape (Tang and Gerlach, 2017), it is possible that in addition to reducing migratory potential, inhibiting ezrin activity impaired the ability of the cells to remain attached to the membrane, possibly through dysregulation of focal adhesions (Hoskin et al., 2015). Due to the reduction in cell numbers in samples treated with NSC668394, we were unable to assess the effect of ezrin inhibition on proliferation using our current methods.

In the face of hypoxia, we found that p-ezrin levels were increased in PASMCs *in vivo* and *in vitro*. The mechanism by which hypoxia might lead to an enhancement of ezrin phosphorylation and activation are unclear. Ezrin phosphorylation at Thr567 can be mediated by several kinases, including Rho kinase and PKC (Matsui et al., 1998). Indeed, numerous studies have shown hypoxia-induced increases in Rho kinase activity in PASMCs (Wang et al., 2001; Fagan et al., 2004; Wang et al., 2005; Weigand et al., 2005), providing a plausible pathway for ezrin activation by hypoxia in this cell type. Further experiments will be required to determine the exact pathway involved in hypoxic enhancement of p-ezrin levels.

A main role of NHE1 is control of pH_i_. Increasing NHE1 expression led to a significant increase in NHE activity, and we previously showed that the elevation in NHE1 protein in hypoxic PASMCs was accompanied by an alkaline shift in pH_i_ (Rios et al., 2005). Changes in pH_i_ have multiple effects on smooth muscle cell function (Krampetz and Rhoades, 1991; Quinn et al., 1996; Madden et al., 2001; Putney et al., 2002; Christou et al., 2012). Thus, it might seem reasonable that the increase in pH_i_ induced by NHE1 overexpression is the driving mechanism for PASMC migration and proliferation. On the other hand, based on our results using a mutant form of NHE1 that cannot bind p-ezrin, but maintains exchanger function, it might be tempting to conclude that changes in pH_i_ are not the main downstream effector of NHE1. To our knowledge, the exchange function of NHE1 that interacts with actin fibers would be similar to non-actin interacting NHE1 as these two functions (ion exchange and ezrin binding) are believed to be independent and require different sections of the protein, with the ion translocation region located in the middle of the protein (residue 262) and the ezrin-binding site located in the cytosolic C-terminal tail region (residues 500–560). Previous work has shown that disrupting ion translocation does not prevent actin binding (Denker and Barber, 2002), and we show that preventing ezrin/actin binding does not alter ion exchange activity (Figure 4). While revealing an important component of NHE1-induced regulation of cell function, it is important to note that our experiments do not rule out a synergistic, or even required, role for changes in pH_i_, which on their own may not be sufficient to change cell phenotype. Another remaining question is whether NHE1 localizing along actin fibers creates pH microdomains which might exert a localized influence on signaling. Future experiments using a mutant form of NHE1 lacking exchange activity, but still maintaining p-ezrin interactions (Denker and Barber, 2002) would help to solve these questions.

While our results show that p-ezrin activation and NHE1/SMA interactions are required for both migration and proliferation in PASMCs, the downstream mechanisms mediating these effects could be different. Previous studies showed p-ezrin directly promotes cell mobility and migration in cancer cell lines through the activation of different signaling pathways, including Rac1, CD44 and Cdc42 (Gautreau et al., 1999; Zohar et al., 2000; Prag et al., 2007; Saito et al., 2013), while in pancreatic cancer ezrin activates FAK/Akt to promote proliferation (Xu and Zhang, 2021). Whether the interactions between p-ezrin, NHE1 and actin, for example as a scaffolding complex, might bring signaling molecules in close proximity, or perhaps create localized pH microdomains that are required for activation of these downstream pathways, is unclear. Via regulation of cell stiffness, actin polymerization or cytoskeletal regulation, NHE1/actin interactions might also contribute to promoting cell movement or shape change, both of which are required for migration and proliferation. For example, recent work in *Drosophila* indicates a role for ezrin-mediated actin/plasma membrane tethering in mitotic rounding (Leguay et al., 2022), suggesting a mechanical mechanism by which NHE1/ezrin/actin interactions could potentially modulate proliferation. Further experiments will be needed to delineate the exact mechanisms by which the proposed pathway control migration and proliferation.

In summary, we describe a mechanism by which interactions between NHE1, p-ezrin, and SMA lead to changes in PASMC function. Under conditions of hypoxia, where p-ezrin, and at later time points, NHE1 protein levels are increased, these interactions appear to facilitate changes in PASMC phenotype that would promote vascular remodeling and the development of pulmonary hypertension. Because of the ubiquitous nature of NHE1 and its critical role in regulating intracellular pH, inhibiting or silencing this protein is not a feasible therapeutic treatment for patients. Similarly, our finding that the ezrin inhibitor caused substantial PASMC death indicates this protein is also not a suitable target. Instead, understanding the specific downstream mechanism by which NHE1 exerts its effects on cell function, particularly one that can be separated from ion exchange activity (i.e., targeting the NHE1/ezrin interaction site), may provide new pathways to explore for drug development.

## Data Availability

The original contributions presented in the study are included in the article/supplementary materials, further inquiries can be directed to the corresponding author.
